# Investigating the Structure of the French WISC–V (WISC–V^FR^) for Five Age Groups Using Psychometric Network Modeling

**DOI:** 10.3390/jintelligence11080160

**Published:** 2023-08-10

**Authors:** Thierry Lecerf, Salome Döll, Mathilde Bastien

**Affiliations:** 1 Faculty of Psychology and Educational Sciences, University of Geneva, 1205 Geneva, Switzerland; salome.doll@unige.ch (S.D.); mathilde.bastien@unige.ch (M.B.); 2 Institute of Psychology, Faculty of Social and Political Sciences, University of Lausanne, 1015 Lausanne, Switzerland

**Keywords:** WISC–V^FR^, psychometric network analysis, exploratory graph analysis (EGA), bootEGA, construct validity, dimensionality, intelligence, age

## Abstract

Since the seminal work of Spearman, exploratory and confirmatory factor analysis represents the standard method of examining the dimensionality of psychological instruments. Recently, within the network psychometrics approach, a new procedure was proposed to estimate the dimensionality of psychological instruments: exploratory graph analysis (EGA). This study investigated the structure of the French Wechsler Intelligence Scale for Children–Fifth Edition (WISC–V^FR^) with five standardization sample age groups (6–7, 8–9, 10–11, 12–13, and 14–16 years) using EGA. The primary research questions include (a) how many WISC–V^FR^ dimensions are identified in each age subgroup? (b) how are subtest scores associated within the dimensions? Because the number and the content of the dimensions identified by EGA could vary with samples, the secondary research questions include (c) is there evidence of reproducibility and generalizability of the dimensions identified by EGA? We used another procedure called bootstrap exploratory graph analysis (bootEGA). EGA and bootEGA suggested only three dimensions, which are consistent with processing speed, verbal comprehension, and the “old” perceptual reasoning factor. Results did not support the distinction between visual–spatial and fluid reasoning dimensions. EGA and bootEGA represent new tools to assess the construct validity of psychological instruments, such as the WISC–V^FR^.

## 1. Introduction

Since the pioneering work of Galton and Binet (with Simon), the main purpose of differential psychology is to describe and explain [inter]-individual differences, and, to a lesser extent, intra-individual variability with tests and theories. The main issue regarding test development and psychological [intelligence] theory is to estimate the correct number of dimensions/factors^1^ underlying individual differences. To deal with this goal, Spearman (1904, 1927) developed factor analysis to investigate the covariance structure between variables (French, English, etc.). The most robust finding of Spearman (1904, 1927) was the observation of positive correlations between all cognitive variables. Individuals with better performance in one variable also tend to have better performance in other variables. This phenomenon was called the *positive manifold*, which is a well-known and the most replicated phenomenon within intelligence studies (Schmank et al. 2019; Troche et al. 2021; van der Maas et al. 2006). Spearman adopted a reflective conception of intelligence and considered that the positive manifold reflects a common source of variation, an unobservable factor (i.e., a latent variable), that causes performances in all cognitive variables: the general factor of intelligence (labeled the *g*-factor). It means that variables are locally independent from each other because covariance among variables is only and fully explained by the latent variable, the *g*-factor. However, in 1916, this causal psychological interpretation of the *g*-factor was challenged by Thomson. He hypothesized that the *g*-factor does not necessarily reflect a psychological attribute, but that the pattern of overlapping psychological attributes causes the positive manifold (Bartholomew et al. 2009). Thomson proposed a “bonds”^2^ or a “sampling” model of intelligence, and stated (Thomson 1916, p. 272) that “…*an excellent hierarchy can be made with Specific and Groups factors only, without a General Factor*”. According to Thomson, the *g*-factor is not a psychological construct, but a statistical one. A distinction should be made between “psychometric *g*” and “psychological *g*”.

Exploratory factor analysis (EFA) and confirmatory factor analysis (CFA) are commonly used to investigate the internal structure of psychological instruments (Benson et al. 2010; Dombrowski et al. 2017; Golino and Demetriou 2017; Golino and Epskamp 2017; McFarland 2019; McGrew 2023; McGrew et al. 2023; Schmank et al. 2019; Wilhelm and Kyllonen 2021). Hence, EFA and CFA are related to the development of tests and of psychological [intelligence] theory. Within EFA, several procedures are proposed to estimate the correct number of dimensions (i.e., factors; Watkins 2021): the Kaiser–Guttman eigenvalue-rule (i.e., eigenvalues ≥1), Horn’s parallel analysis (HPA; Horn 1965), the minimum average partial procedure (MAP, Velicer 1976), very simple structure (VSS), the visual scree-test, the standard error of scree (SEscree), and indices such as BIC and/or EBIC, etc. Nevertheless, all criteria have their owns limits (Golino and Epskamp 2017; Golino and Demetriou 2017). It should be mentioned that while the first issue with EFA concerns the [correct] number of dimensions, the second one relates to the type of rotation that must be used (none, orthogonal, oblique).

To overcome some limitations inherent in EFA, Golino and Epskamp (2017) have developed an alternative method to examine the dimensionality of psychological instruments and to estimate the correct number of dimensions: *exploratory graph analysis* (EGA) (Golino and Demetriou 2017; Golino et al. 2020; 2022). EGA is an alternative data-driven method that belongs to what is called the “network psychometrics” approach (Bulut et al. 2021; Borsboom and Cramer 2013; Epskamp et al. 2017; Epskamp et al. 2018a, 2018b; Golino and Demetriou 2017; Isvoranu et al. 2022; Marsman and Rhemtulla 2022). With network psychometrics (NP), the main goal is to explain the structure of the covariance between observed variables by causal, reciprocal interactions between these observed variables. NP theory conceptualizes a psychological attribute as a set of interconnected components and estimates the relations among those components (Isvoranu et al. 2022). It means that a psychological phenomenon represents an emergent property arising from the relations among those components. This theoretical perspective is founded on graph theory, the philosophy of psychiatry, network science (e.g., dynamical systems theory, catastrophe theory, cybernetics), systems science, and on the need to adopt a more complex perspective of psychological attributes (Henry et al. 2022; Isvoranu et al. 2022; Robinaugh et al. 2020). Within the NP approach, the psychological variables (items, tests, etc.) are called *nodes* (vertices), and statistical relations between pairs of nodes are called *edges* (links; Johal and Rhemtulla 2021; Kan et al. 2019). The statistical relations are estimated (not observed). An edge’s thickness indicates the strength of the relationship between the nodes (a thicker edge indicates a stronger relationship), and an edge’s color indicates the direction of the correlation (blue or green for positive correlations, and red for negative correlations). This type of model represents undirected graphical models, called *pairwise Markov random field* (PMRF). Based on strategies used in the network science to analyze network structure, local network properties can be quantified with centrality, the clustering of nodes, and global network properties, such as the small-worldness of a network.

Within psychology, the development of network psychometric approaches is indebted to Peter Molenaar, who understood the similarity between the Ising model introduced in statistical physics for studying magnetic interactions and item response theory within psychometrics models (Borsboom 2022; Epskamp et al. 2018b; Isvoranu et al. 2022; Marsman and Rhemtulla 2022). Molenaar is also well-known for having popularized the notion of ergodicity within psychology. According to Molenaar, the Ising model, which corresponds to the Markov random field for binary data, is equivalent to the Rasch model (1PL). More precisely, the Ising model is equivalent to logistic regression models, log-linear models, and the multivariate two-parameter logistic model (MIRT; Epskamp et al. 2018b). However, while the Rasch model represents a latent variable model with a reflective measurement model of interpretation, with NP there is no causally latent variable. Historically, the first step of the network approach was the network visualization, the “discovery of network structure” (Marsman and Rhemtulla 2022). Several years later, new procedures were developed to assess the robustness of networks, and to quantify the uncertainty of the network parameters. Currently the statistics procedures for networks are mainly exploratory. Confirmatory methods are in progress. One current limit with the NP approach is related to the interpretation and use of network models. Developing formal theories is necessary (Isvoranu et al. 2022; Henry et al. 2022; Robinaugh et al. 2020).

While NP was mainly applied to the field of psychopathology (Borsboom 2008; Henry et al. 2022; Marsman and Rhemtulla 2022), it has been seldom applied in the field of intelligence. As far as we know, within the domain of intelligence, the only developmental model that fits into the NP approach is the dynamical mutualism model developed by van der Maas et al. (2006, 2017, 2019)^3^. This model revolves around the Lokta–Volterra mutualism model (Savi et al. 2021). According to this mutualism model, the positive correlations among variables (i.e., the positive manifold) are explained by positive reciprocal causal interactions between different cognitive processes and abilities across the development. As in Thomson’s model (Thomson 1916), the general factor of intelligence does not correspond to a psychological attribute but emerges from the dynamic interactions between cognitive abilities. In this model, the basic cognitive abilities are independent at birth, and positively interact during the development. It means that correlations between cognitive abilities will increase during development, rather than decreasing as suggested by Cattell (Horn and Cattell 1967) and the differentiation hypothesis (Garrett 1946; Lecerf and Canivez 2018).

Like EFA, the main goal of NP is to describe and to explain interindividual differences in psychological instruments, by modeling the variance–covariance structure of the variables, that is, between nodes. However, there are several substantial differences between EFA and NP (Kan et al. 2019). The most important one concerns the explanation of the covariance between variables. EFA is a causal latent variable model: observable variables (manifest variables) are related together through common, unobserved variables (latent variables; Jordan et al. 2020). This is a reflective, causal approach^4^. A common source of variation explains the positive covariation between variables (Christensen et al. 2020). In contrast, NP theories do not postulate the presence of latent variables^5^. This approach assumes that some variables may correlate more together than with other variables, and hence, can form “clusters” of measure, because some variables may share more common variance. Cluster (or community) is defined as a set of connected nodes (Golino and Epskamp 2017; McFarland 2020). Clusters of nodes represent dimensions and are equivalent with latent variables in EFA and/or CFA (Christensen and Golino 2021a). Within the NP approach, new procedures were developed to examine the dimensionality of psychological instruments (i.e., EGA) and for understanding psychological attributes, without [necessarily] postulating the existence of latent variables (Kan et al. 2020).

A second difference between EFA and NP concerns the measure of associations used to estimate parameters. While EFA uses zero-order correlations, some network models, as it is the case for EGA, are based on partial correlations (Isvoranu et al. 2022; McFarland 2020; Schmank et al. 2021). Partial correlation means that conditional dependencies between two subtest scores are estimated after controlling for the association of these two subtest scores with all other subtest scores in the network (Epskamp and Fried 2018; Isvoranu et al. 2022). In other words, edges represent partial correlation coefficients between two variables. If a partial correlation coefficient is exactly zero between two nodes, it means that there is no association between these two nodes after controlling for all other associations, thus after removing the general variance. Remember that NP estimates the direct relations between observed variables, without postulating latent variables. Within the NP approach, EGA was developed by Golino and Epskamp (2017) with the main goal to examine the dimensionality of psychological instruments, and hence to determine the correct number of dimensions. By focusing on the unique variance between pairs of variables (partial correlations) instead of the variance shared by all variables, EGA indicates which subtest scores belong to each dimension/cluster by using community detection. EGA allows the visualization of the relations between variables via a network plot. Using simulation data, Golino and Epskamp (2017; Golino and Demetriou 2017) suggested that EGA is comparable, or even more accurate, than principal axis factoring (PAF) with the Kaiser–Guttman rule (eigenvalue >1), HPA, MAP, EBIC, EBIC, and VSS to determine the correct number of dimensions. Golino and Epskamp (2017) simulated 32,000 data sets with known factor structures. Simulation data varied with the number of factors (2 and 4), the number of items (5 and 10), the sample size (from 100 to 5000), and the correlation between factors (0.00, 0.20, 0.50, and 0.70). Sixty-four conditions were created with 500 simulated data sets for each condition. When the number of factors was 2, EGA performed similarly with HPA, EBIC. EGA performed better than EFA methods with 4 factors, when the correlations between factors was 0.70, with a small number of items per factor (5), and when the sample size was large (5000). EGA also outperformed EFA methods with 5 items per factor and with high correlations between factors. In sum, with 4 factors and correlations between factors equal to 0.50, EGA outperformed the EFA methods, irrespective of the sample size, and the number of items per factor. EGA was less affected by all variations.

By using EGA, the main objective is to determine which edges must be included in the network, and to select the best model. Model selection is the most fundamental issue because it is necessary to remove “non-significant” edges and to keep only “true” edges.

Another important question regarding the dimensionality of psychological instruments and the number of dimensions identified by EGA is related to the stability of the network dimensions (Isvoranu et al. 2022). Indeed, the variability of samples or parameter estimation can lead to inconsistency in the number of dimensions and in the content of each dimension identified with EGA. To estimate the reproducibility and the stability of the network, a new bootstrap approach has been developed: *bootstrap exploratory graph analysis* (bootEGA; Christensen and Golino 2021b). On the one hand, bootEGA introduces a bootstrap procedure to estimate the stability and the consistency of the dimensions identified by EGA, and allows the determination of whether EGA’s dimensions are stable across replicate samples or whether the dimensional configurations change across replicate samples. Furthermore, bootEGA estimates the stability of the dimensions by structural consistency, that is, the proportion of time that each dimension of EGA is exactly retrieved across samples. The structural consistency implies that the composition of each dimension is strictly the same. We can consider that the structural consistency corresponds to the estimation of internal consistency within EFA, and hence can be interpreted as an indicator of the homogeneity of the dimension (Christensen et al. 2020).

On the other hand, bootEGA estimates the stability of each subtest score within each dimension, by using a bootstrap approach on the EGA results. Item stability corresponds to the proportion of time a subtest score is replicated in the same dimension across samples. Based on Christensen and Golino’s (2021b) suggestion, we consider that a subtest score is correctly placed within a dimension when the value is ≥0.70. Christensen and Golino (2021b) showed that EGA and bootEGA provide a similar estimation of the number of dimensions, but that bootEGA is better than EGA for the placement of subtest scores within the dimensions.

To the best of our knowledge, few studies have conducted NP analysis with intelligence tests (Golino and Demetriou 2017; Kan et al. 2019; McGrew et al. 2023; Schmank et al. 2019). One study was conducted on the WAIS–III (van der Maas et al. 2017), two on the WAIS–IV (Kan et al. 2019; McFarland 2020; Schmank et al. 2019), and two on the Woodcock–Johnson IV (Bulut et al. 2021; McGrew et al. 2023). Schmank et al. (2019) conducted NP analysis and confirmatory factor analysis on the Hungarian WAIS–IV. Results disclosed four to five clusters of nodes: working memory (Gwm), processing speed (Gs), comprehension–knowledge (Gc). The distinction between fluid reasoning (Gf) and visual processing (Gv) was not very clearly supported. Matrix Reasoning and Figure Weights were strongly connected with Block Design, Visual Puzzles, and Picture Completion, which makes sense in the “old” perceptual reasoning factor. Schmank et al. (2019) found that working memory and fluid reasoning subtest scores are more central in the network. In addition, they showed that for the Hungarian WAIS-IV, the NP model was better than the classical higher-order model. However, they suggested that the choice between latent variable models and network psychometrics depends on the intelligence theory. The *g*-factor theory is compatible with latent variable model, while mutualism theory is more compatible with NP. Bulut et al. (2021) applied NP to the WJ IV COG to test invariance across sex and age groups. They showed that the network structure was the same for all age and sex groups. Instead of using multigroup CFA to test measurement invariance (Dombrowski et al. 2021), Bulut et al. (2021) used NP. Results provided evidence for the usefulness of the NP. Kan et al. (2019) compared latent variable models and NP models of the WAIS-IV variance–covariance matrix. Results indicated that the NP model provides a better fit to the data than the latent variable model. According to Kan et al. (2019), this finding suggests that the mutualism model of van der Maas et al. is a better explanation of the variance–covariance structure than the higher-order model. In other words, the hypothesis of a common source of variation, a *g*-factor, is not necessary to account for the correlation between variables. Reciprocal dynamic interactions between different cognitive processes and abilities are sufficient to explain the correlations between variables, the positive manifold. From this perspective, the *g*-factor is an emergent property. Finally, McGrew et al. (2023) conducted NP on the WJ-IV with a school age sample. They found a model with eight dimensions: Gf, Gc, Gv, Gwm, Ga, Gr, Gs, and Gq. This study also demonstrated that NP can be useful to examine the dimensionality of psychological instruments.

Based on the variance–covariance matrix among subtest scores, the structure of the WISC–V^FR^ was established by the publisher through confirmatory factor analysis only (CFA; Wechsler 2016). However, many changes have been introduced between the WISC–V^FR^ and the WISC–IV^FR^. The publisher favors a higher-order model with five first-order factors: verbal comprehension (VC), visual–spatial (VS), fluid reasoning (FR), working memory (WM), and processing speed (PS). The WISC–V^FR^ publisher indicates that this factor structure is appropriate for the total sample (N = 1049) and for the five age groups. However, exploratory factor analyses (EFA) conducted by Lecerf and Canivez (2018) on the WISC–V^FR^ total standardization sample, and by Lecerf and Canivez (2022) on the WISC–V^FR^ five age standardization samples (6–7, 8–9, 10–11, 12–13, and 14–16 years) do not support a five-factor model. Whether for the total sample or the five age groups, results are more consistent with a four first-order factors model. Their results do not support the distinction between visual–spatial and fluid reasoning. These findings are consistent with those obtained for the US WISC–V (Canivez et al. 2018; Dombrowski et al. 2018, 2021), the UK WISC–V (Canivez et al. 2019), the German WISC–V (Pauls and Daseking 2021), and the Scandinavian WISC–V (Egeland et al. 2022).

Thus, unlike previous studies testing the internal validity of the Wechsler Intelligence Scale with EFA and/or CFA, the first purpose of this study is to apply EGA to examine the dimensionality of the WISC–V^FR^ for five standardization sample age groups (6–7, 8–9, 10–11, 12–13, and 14–16 years). As far as we know, EGA was never applied to the five WISC–V^FR^ standardization sample age groups. By using EGA, the main objective is to determine which edges must be included in the network and to select the best model. Our second goal is to use bootEGA to estimate the stability of the dimensions identified by EGA and to estimate the stability of each subtest score within a dimension (Christensen and Golino 2021b). Determining which dimensions and which subtest scores are stable is very important and can improve the clinical utility of the scores of the WISC–V^FR^, and hence, the construct validity of the WISC–V^FR^. Based on Hunsley and Mash (2007), we defined clinical utility as the extent to which test scores allowed the better understanding of the cognitive functioning of the child, and includes diagnostic utility and decision making (Canivez et al. 2019). Clinical utility is related to construct validity because construct validity pertains to the interpretation of test scores (Hughes 2018; see Borsboom et al. 2004 for an alternative view of validity). The *Standards for Educational and Psychological Testing* (AERA 2014) defined validity as: are you measuring what you think you are measuring, and are your measures useful for decision making? Construct validity includes five different sources of evidence, which all assess construct validity. Internal validity is one of these five sources and is a necessary but not a sufficient condition for clinical utility (Hughes 2018). By using exploratory analyses, like EGA and bootEGA, we will provide useful and complementary information about the structure and the construct validity of the WISC–V^FR^ and the generalizability of dimensions and subtest scores in a dimension (McGrew 2023). To our knowledge, this study is the first one to use EGA (partial correlations) to examine the structure of the WISC–V^FR^ for the five age groups.

As mentioned before, the publisher of the WISC–V^FR^ provided evidence of the factorial structure of the WISC–V^FR^ with confirmatory factor analyses only (CFA); exploratory analyses were not conducted. However, the WISC–V^FR^ deviates from the WISC–IV^FR^: three subtests were added in the new WISC–V^FR^ (FW, PS, VP), and two subtests were removed (Picture Concepts, Word Reasoning). Substantive structural and methodological changes were introduced in the WISC–V^FR^. Therefore, and as recommended by some methodologists, the assessment of the structural validity of a new test or a new version of tests, should be conducted with exploratory procedures in complement with confirmatory analyses; a revised test should be treated as a new one (Beaujean 2015; Gorsuch 1983). Accordingly, the purpose of this study was to examine the structural validity of the WISC–V^FR^ with exploratory procedures, EGA and bootEGA, to support the interpretation and the use of the WISC–V^FR^ subtest scores.

## 2. Materials and Methods

### 2.1. Participants

The summary statistics for each age group (i.e., correlations) reported in the WISC–V^FR^ interpretive manual were used to conduct EGA and bootEGA analysis (Wechsler 2016). The total French standardization sample includes 1049 children-adolescents and is representative of the population of France in terms of age, sex, parental education levels, and geographic regions (i.e., general census of the population made by the INSEE in 2010). Eleven age groups were created from 6 years to 16 years and 11 months. Each age group is composed of 80 to 104 children, and the number of girls and boys is balanced in each age group. The standardization sample was obtained using six parent education levels (no degree (14%); ≤ 12 years (7%); 13 years (25%); 15 years (18%); 17 years (15%), ≥18 years (21%)), and according to five geographic distributions (Parisian (26%), northwest area (27%), northeast (20%), southeast (17%), and southwest areas (10%)). Five correlation matrices were used to represent five broad age subgroups. Each age subgroup is composed of 181 to 263 children (6–7 [n = 201], 8–9 [n = 204], 10–11 [n = 200], 12–13 [n = 181], and 14–16 [n = 263]). Because we used the correlation matrices reported in the WISC–V^FR^ interpretive manual, ethical review and approval were not necessary for this study.

### 2.2. Instrument

The WISC–V^FR^ is a standardized, individual test of intelligence for children and adolescents (6 to 16:11 years old). It consists of 15 subtests. The Full-Scale IQ [FSIQ] estimates general intelligence and is based on the sum of 7 primary subtests: Similarities (SI), Vocabulary (VO), Matrix Reasoning (MR), Figure Weights (FW), Block Design (BD), Digit Span (DS), and Coding (CD). To estimate the five primary indexes, three subtests are necessary in addition to the 7 subtests mentioned above: Visual Puzzles (VP), Picture Span (PS), and Symbol Search (SS). Based on the CHC *compendium* of cognitive abilities (Schneider and McGrew 2018), the five primary indexes are: Verbal Comprehension [*Gc*–VC: SI, VO], Visual Spatial [*Gv*–VS: BD, VP], Fluid Reasoning [*Gf*–FR: MR, FW], Working Memory [*Gwm*–WM: DS, PS], and Processing Speed [*Gs*–PS: CD, SS]. The WISC–V^FR^ includes five secondary subtests: Information (IN), Comprehension (CO), Letter-Number Sequencing (LN), Cancellation (CA), and Arithmetic (AR). The FSIQ and the five indexes are standard scores (*M* = 100, *SD* = 15). The subtest scores are also standardized scores (*M* = 10, *SD* = 3). In addition, five ancillary index scores are also available: Quantitative Reasoning [QR], Auditory Working Memory [AWM], Nonverbal [NV], General Ability [GA], and Cognitive Proficiency [CP].

As reported in the technical manual of the WISC–V^FR^, internal consistency of subtest scores ranged from 0.77 (CO) to 0.94 (FW). Only two of the fifteen internal consistency coefficients were lower than 0.80 (CO and SS). The internal consistency of indices ranged from 0.87 (PSI) to 0.95 (FSIQ). Three internal consistency coefficients were lower than 0.90 (VCI, WMI, PSI).

The correlation coefficients ranged from 0.04 (FW-CA) to 0.66 (SI-VO) for 6–7 years, from 0.05 (SI-CA) to 0.63 (SI-VO) for 8–9 years; from 0.10 (FW-CA) to 0.70 (SI-VO) for 10–11 years; from 0.00 (VO-CA) to 0.65 (SI-IN) for 12–13 years; and ranged from 0.10 (VO-CA) to 0.58 (SI-VO) for 14–16 years.

### 2.3. Analyses

Exploratory NP analyses were conducted on the five correlation matrices reported in the WISC–V^FR^ interpretive manual. Because we used the data reported in the WISC–V^FR^ *interpretive manual*, we know that the quality of the data is relatively appropriate for network analysis (i.e., measurement error). Furthermore, the positive manifold is strong enough in the WISC–V^FR^ to use EGA and bootEGA. All network analyses were completed using R (R Core Team 2022) and RStudio version 2022.12.0 + 353. Network analyses were performed using the R-packages EGAnet (1.2.4) and networktools (1.4.2). Guidelines provided by Isvoranu et al. (2022); Epskamp and Fried (2018); Epskamp et al. (2018a) were followed.

EGA provides a Gaussian graphical model (GGM) which is based on partial correlations. According to Christensen and Golino (2021b; Golino et al. 2022), we applied EGA with GLASSO, and a Walktrap community detection algorithm. EGA was applied on each replicate sample until the intended number of samples, which is 500 in the present study. By using EGA, the main objective is to determine which edges must be included in the network and to select the best model. Model selection is the most fundamental issue because it is necessary to remove “non-significant” edges and to keep only “true” edges. Model selection is based on the most important criteria, which are sensitivity (identification of “true” edges) and specificity (identification of “true” negative). EGA uses a model selection algorithm based on statistical regularization (i.e., penalized likelihood estimation vs. unregularized estimation methods like thresholding, pruning, or model selection). In a first step, EGA starts with the correlation matrix of observed variables. Next, EGA inverts the variance–covariance matrix to obtain the partial correlation matrix (i.e., precision matrix). Each element of the precision matrix corresponds to an edge, which can be interpreted as the partial correlation coefficient between two variables after controlling for the association of these two subtest scores with every other subtest scores in the network. Then, because two conditionally independent variables could have an estimated partial correlation that is nonzero, the next step is to shrink low partial correlations to zero (i.e., “pruning” as removal of spurious estimated edges) to generate sparse networks. Sparsity is imposed to the precision matrix (Epskamp and Fried 2018). One classical method used to introduce sparsity involves the Graphical Least Absolute Shrinkage and Selection Operator regularization (GLASSO; Christensen and Golino 2021b). With GLASSO, the amount of penalization is chosen based on the tuning hyperparameter (λ), which shrinks edges to zero (lower values of λ remove few edges)^6^. EGA computes models with approximately 100 different values of λ, and the selected model is the one that minimizes EBIC (*extended Bayesian information criterion*). EBIC relies on a hyperparameter (γ) that controls for the simplicity of the network. Higher γ values lead to sparser models. EGA starts with γ = 0.50. Finally, the Walktrap algorithm (community detection algorithm) is used to identify the number and the content of dense subgraphs of the sparse inverse correlation matrix (communities), and to examine the number of dimensions (Christensen and Golino 2021b). In sum, EGA estimates the [correct] number of dimensions by using GLASSO (i.e., penalized maximum likelihood estimation) and a walktrap algorithm (random walk algorithm). As mentioned before, Golino and Epskamp (2017) showed, using simulation data, that EGA with GLASSO is comparable or more adequate than EFA to examine the dimensionality of psychological instruments.

Second, bootEGA allows the examination of the stability of EGA’s results. Because bootEGA was conducted on correlation matrices, we used a parametric procedure. bootEGA provides a sampling distribution of EGA results and reports several descriptive statistics: the median number of dimensions based on the median value of each edge across the replicate samples, the standard deviation of the number of dimensions, a confidence interval, and a percentile bootstrap confidence interval. bootEGA also provides a typical network. Furthermore, bootEGA estimates the stability of the dimensions by *structural consistency*, that is, the proportion of time that each dimension of EGA was exactly retrieved across the replicate bootstrap samples. Structural consistency corresponds to the estimation of internal consistency within EFA, and hence, can be interpreted as an indicator of the homogeneity of the dimension (Christensen et al. 2020). To our knowledge, no recommendations for what a “high” structural consistency is was given. Because we consider that cognitive broad abilities must be homogenous to allow substantive interpretation, we choose a value of 0.75 or higher. It means that the dimension replicates across ≥75% of the bootstrap samples.

bootEGA also reports *item stability*, which estimates the strength of each variable within a specific dimension, a specific cluster. Item stability indicates the proportion of time each subtest score is placed within a specific dimension. Thresholds values are provided by Christensen and Golino (2021b) and represent the optimal balance between false positives and negatives (i.e., sensitivity and specificity). According to these authors, item stability values ≥0.70 are considered stable. In brief, we used bootEGA to estimate the stability of the dimensions of the WISC–V^FR^ identified by EGA for the five age groups.

## 3. Results

### 3.1. Ages 6 to 7 Years Network Analyses

EGA with the GLASSO estimation and Walktrap community detection algorithm estimated three clusters of nodes, three dimensions (see Table 1). A first dimension represents a mixture of visual–spatial ([VS]; BD, VP), fluid reasoning ([FR]; MR, FW, AR), and two working memory subtest scores ([WM]; DS, PS). The second dimension represents verbal comprehension ([VC]; SI, VO, IN, CO) with Letter–Number Sequencing (LN), while the third dimension is consistent with processing speed ([PS]; CD, SS, CA).

Parametric^7^ bootEGA with 500 iterations for the age group 6–7 years estimated a typical (median) structure with three dimensions, which is exactly the structure estimated by EGA. Thus, the first dimension is a mixture of visual–spatial, fluid reasoning, and working memory, while the second dimension represents verbal comprehension with Letter-Number Sequencing. The third dimension is consistent with processing speed (CD, SS, CA).

Then, we estimated the stability of the dimension solution, by looking at the frequency of each dimension solution. Frequencies indicate that three dimensions were found 55% of the time of bootstrap replicates, four dimensions were found 29.2% of the time, and two dimensions were found 10% of the time. A five dimensions structure was found 2.4% of the time. These results suggest that the three dimensions solution was relatively unstable.

Next, the structural consistency of each dimension was estimated. Structural consistency allows the estimation of how often each dimension is the same across the replicate sample (i.e., identical subtest scores placement). Results indicate that all dimensions were very unstable (dimension 1[ FR-VS-WM] = 24.6%; dimension 2 [VC + LN] = 47.6%, and dimension 3 [PS] = 53.2%). This instability is consistent with the *item stability* analysis. Results indicated that six subtest scores values were below 0.70 and hence unstable (FW, PS, DS, AR, LN, CA), while nine subtest scores were stable (≥0.70: MR, VP, BD, VO, CO, SI, IN, CD, SS). Most of the unstable subtest scores belonged to dimension 1 [FR-VS-WM].

Regarding these unstable subtest scores, the analyses of the item stability values across each dimension in the bootstrap samples indicate that FW was sometimes replicated in dimension 1 ([FR-VS-WM]: 65%) and dimension 2 ([VC]: 21%)^8^; Picture Span (PS) was replicated in dimension 1 (60%), dimension 2 (18.3%), and dimension 4 (which may represents WM; 16.6%); AR was replicated in dimension 1 (54.4%), dimension 4 (23.8%), and dimension 2 ([VC]: 17.3%); DS was replicated in dimension 1 (57%), dimension 4 (23.7%), and dimension 2 (16.5%); LN was replicated in dimension 2 ([VC]: 49%), dimension 4 (26.7%), and dimension 1 (21.5%). Finally, CA was replicated in dimension 3 ([PS]: 55.6%), and dimension 1 (35%). These six subtest scores were multidimensional, and hence, had cross-loadings.

**Figure 1:**
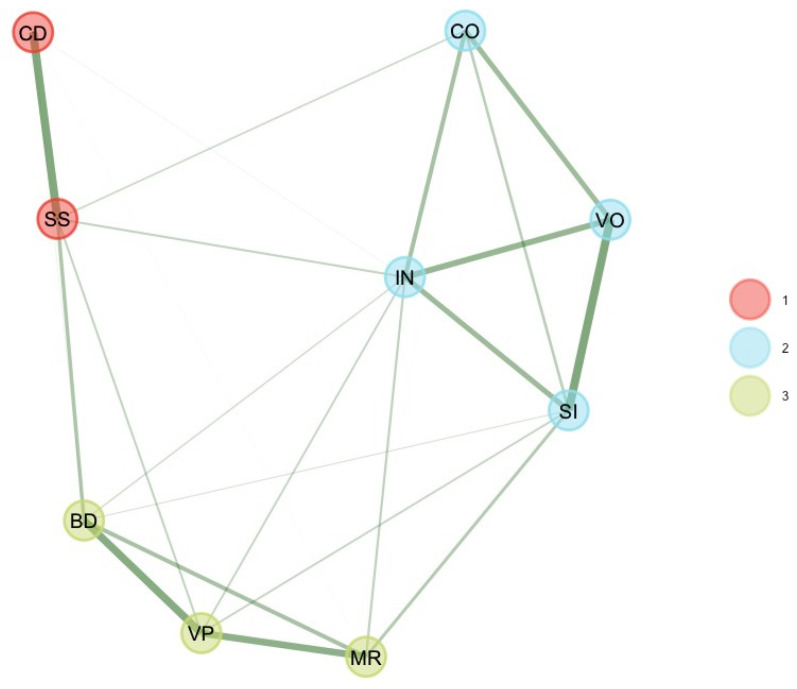
Final bootEGA network for 6–7 years.

As suggested by Christensen and Golino (2021b), these six unstable subtest scores were removed, and EGA and bootEGA were re-estimated. EGA and bootEGA revealed three clusters (see Table 1 and Figure 1): Dimension 1 represents verbal comprehension (SI, VO, IN, CO), while dimension 2 represents the “old” perceptual reasoning factor (BD, VP, MR). The third dimension is consistent with processing speed (CD, SS). We estimated the stability of the dimension solution by looking at the frequency of each dimension solution. Frequencies indicate that three dimensions were found 80.4% of the time of bootstrap replicates, while two dimensions were found 14% of the time of bootstrap replicates. After removing some subtest scores, results indicate that all dimensions were stable (dimension 3 [PS] = 81%) or even very stable (dimension 1 [VC] = 98%; dimension 2 [PR] = 92.6%). This finding is consistent with *item stability* analysis. Item stability values across each dimension in the bootstrap samples indicate that all subtest scores values were higher than 0.70; hence, all subtest scores were stable. Removing these six subtest scores improved the stability of the dimensions.

### 3.2. Ages 8 to 9 Years Network Analyses

EGA with the GLASSO and Walktrap community detection algorithm estimated four dimensions. A first dimension represents a mixture of visual–spatial ([VS]; BD, VP), and fluid reasoning ([FR]; MR, FW), and corresponds to the perceptual reasoning factor. The second dimension represents working memory ([WM]; DS, PS, LN) with arithmetic (AR), while the third dimension is consistent with verbal comprehension ([VC]; SI, VO, IN, CO). Finally, the fourth dimension represents processing speed ([PS]; CD, SS, CA).

Parametric bootEGA for the age group 8–9 estimated a typical (median) structure with the same four dimensions as EGA. Then, we evaluated the stability, by looking at the frequency of each dimension solution. Frequencies indicate that four dimensions were found 54.2% of the time of the 500 bootstrap replicates, three dimensions were found 41.2% of the time, and five dimensions were found 2.8% of the time. These results suggest that the four dimensions solution is relatively unstable.

Next, the structural consistency was estimated. Results indicate that dimensions 3 and 4 were stable (dimension 3 [VC] = 89.8%; dimension 4 [PS] = 90.2%), while dimensions 1 and 2 were unstable (dimension 1 [VS-FR] = 73.6; dimension 2 [WM] = 21.2%). This finding is relatively consistent with item stability analysis, which indicates that four subtest scores values were below 0.70, and hence unstable (LN, DS, PS, AR). The other eleven subtest scores were stable (VP, BD, MR, FW, VO, CO, SI, IN, CD, SS, and CA).

Regarding these four unstable subtest scores, the analyses of item stability values across each dimension in the bootstrap samples indicate that LN was sometimes replicated in dimension 2 ([WM]: 65%) and dimension 1 ([VS-FR]: 25.4%); DS was replicated in dimension 2 ([WM]: 64.4%) and dimension 1 ([VS-FR]: 26.4%); Picture Span (PS) was replicated in dimension 2 ([WM]: 61.1%) and dimension 1 ([VS-FR]: 25.8%); AR was replicated in dimension 1 ([VS-FR]: 69.6%) and dimension 2 ([WM]: 24%). These four subtest scores are multidimensional, and hence have cross-loadings.

**Figure 2:**
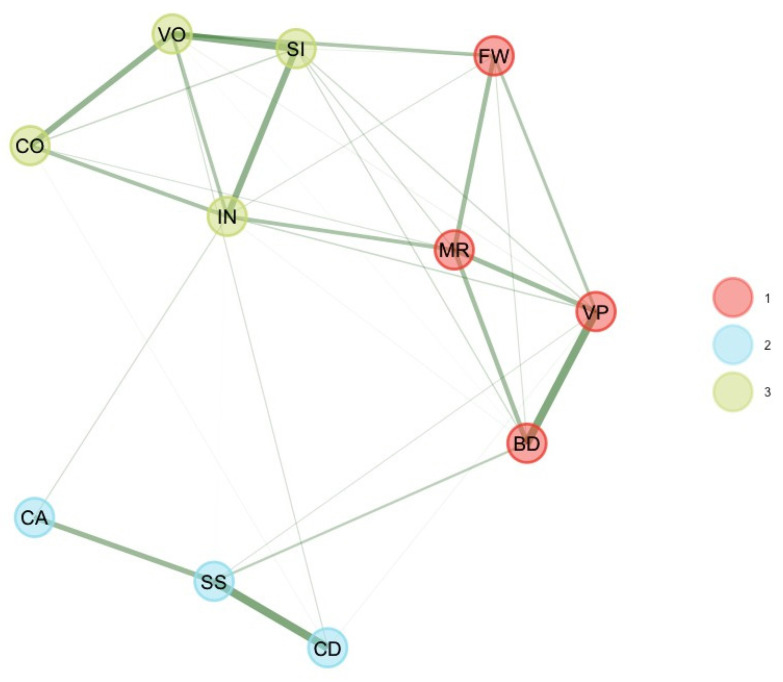
Final bootEGA network for 8–9 years.

These four unstable subtest scores were removed, and EGA and bootEGA were re-estimated. EGA and bootEGA revealed three clusters (see Table 1 and Figure 2): Dimension 1 represents the “old” perceptual reasoning (BD, VP, MR, FW). The second dimension represents verbal comprehension (SI, VO, IN, CO), while dimension 3 represents processing speed (CD, SS, CA). Then, we estimated the stability, by looking at the frequency of each dimension solution. Frequencies indicate that three dimensions were found 95% of the time of bootstrap replicates. Results indicate that all dimensions were relatively stable or very stable (dimension 1 [PR] = 79.2%; dimension 2 [VC] = 94.4%; dimension 3 [PS] = 93.6%). This finding is consistent with item stability analysis. Item stability values across each dimension in the bootstrap samples indicate that all subtest scores values were higher than 0.70; hence, all subtest scores were stable. Removing these four subtest scores improved the stability of the dimensions.

### 3.3. Ages 10 to 11 Years Network Analyses

EGA with the GLASSO and Walktrap community detection algorithm estimated three clusters of nodes, three dimensions. A first dimension represents a mixture of visual–spatial ([VS]; BD, VP), fluid reasoning ([FR]; MR, FW, AR), and two working memory subtest scores ([WM]; DS, LN). Dimension 2 represents verbal comprehension ([VC]; SI, VO, IN, CO) with Picture Span (PS). Dimension 3 is consistent with processing speed ([PS]; CD, SS, CA).

Parametric bootEGA for the age group 10–11 estimated a typical (median) structure with three dimensions, which is relatively like the one estimated by EGA. A first dimension represents verbal comprehension (SI, VO, IN, CO) with Picture Span (PS), while the second dimension represents processing speed (CD, SS, CA). The third dimension represents a mixture of [VS-FR-WM] (BD, VP, MR, FW, AR, DS, LN).

Then, we estimated the stability by looking at the frequency of each dimension solution. Frequencies indicate that three dimensions were found 62% of the time, four dimensions were found 32% of the time, and five dimensions were found 2.6% of the time. These results suggest that the three-dimensions solution was relatively unstable.

Next, the structural consistency was estimated. Results indicate that only dimension 3 [PS] was stable (81.8%). Dimension 1 and dimension 2 [VC-PS] were unstable (respectively, 56.4% and 16.6%). This finding is partially consistent with item stability analysis. Results indicate that all subtest scores were higher than 0.70, and hence stable, except Picture Span (PS). Regarding Picture Span’s score, item stability values across each dimension in the bootstrap samples indicate that it was sometimes replicated in dimension 1 (33%), in dimension 4 (27.6%), and in dimension 2 (25.4%). Thus, Picture Span’s score is multidimensional, and hence has multiple cross-loadings.

**Figure 3:**
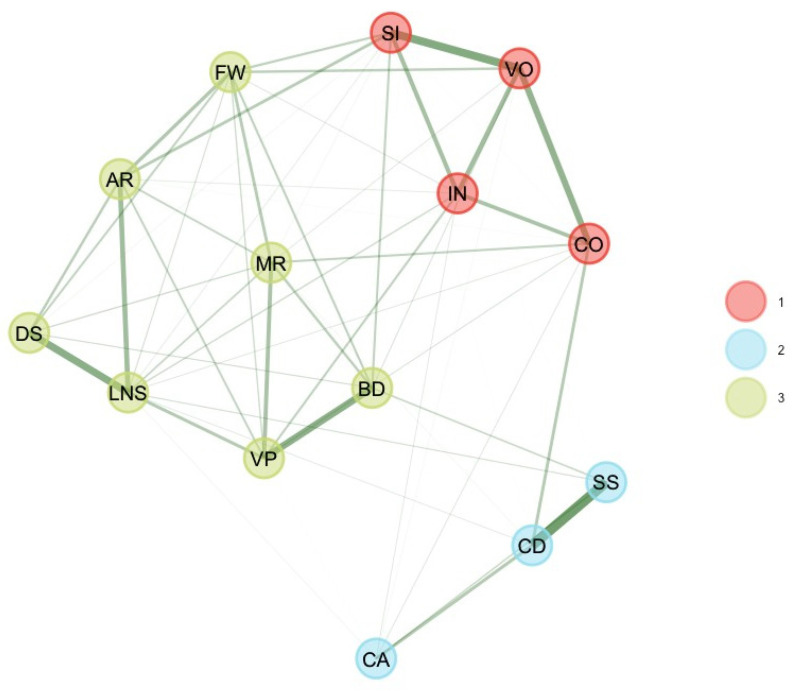
Final bootEGA network for 10–11 years.

Finally, Picture Span’s (PS) score was removed, and EGA and bootEGA were re-estimated. EGA and bootEGA disclosed three clusters (see Table 1 and Figure 3): the first dimension represents verbal comprehension (SI, VO, IN, CO), while the second dimension represents processing speed (CD, SS, CA). The third dimension is a mixture of visual–spatial (BD, VP), fluid reasoning (MR, FW, AR), two working memory subtest scores (DS, LN). Then, we estimated the stability by looking at the frequency of each dimension solution. Frequencies indicate that three dimensions were found 68.6% of the time of bootstrap replicates and four dimensions were found 25.4% of the time of bootstrap replicates. A five-dimension structure was found 2.4% of the time of bootstrap replicates. Furthermore, results indicate that dimension 3 [PS] and dimension 2 [VC] were relatively stable (respectively, 83.2% and 77.2%). Dimension 1 [VS-FR-WM] was still unstable (59%). However, item stability values across each dimension in the bootstrap samples indicate that all subtest scores values were higher than 0.70; hence, all subtest scores were stable. Although item stability was higher than 0.70, the structural consistency of dimension 1 was still inappropriate.

### 3.4. Ages 12 to 13 Years Network Analyses

EGA with the GLASSO and Walktrap community detection algorithm estimated three dimensions (Table 1). A first dimension represents a mixture of visual–spatial ([VS]; BD, VP), fluid reasoning ([FR]; MR, FW, AR), and Picture Span (PS). Dimension 2 represents a mixture of verbal comprehension ([VC]; SI, VO, IN, CO) and working memory ([WM]; DS, LN). Dimension 3 is consistent with processing speed ([PS]; CD, SS, CA).

Similarly, parametric bootEGA for the age group 12–13 reported a network with the same three dimensions: verbal comprehension (SI, VO, IN, CO) with two working memory subtests (DS, LN); a second dimension with fluid reasoning (MR, FW, AR), visual–spatial (BD, VP), and Picture Span (PS). The third dimension is consistent with processing speed (CD, SS, CA). Frequencies indicate that three dimensions were found 81.8% of the time, four dimensions were found 12.8% of the time, and five dimensions were found 1.6% of the time. These results suggest that the three clusters solution was relatively stable.

Regarding the structural consistency, results indicate that only dimension 3 [PS] was very stable (99.4%). Dimensions 1 and 2 were unstable (respectively, 37% and 46.2%). Item stability analysis indicates that three subtest scores values were below 0.70, and hence unstable (LN, FW, AR). The other twelve subtest scores were stable (VP, BD, MR, DS, PS, VO, CO, SI, IN, CD, SS, CA).

Concerning the three unstable subtest scores, item stability values across each dimension in the bootstrap samples indicate that AR was replicated in dimension 1 ([VS-FR]: 68%) and dimension 2 ([VC-WM]: 22.4%); FW was replicated in dimension 1 ([VS-FR]: 68%) and dimension 2 ([VC-WM]: 23.6%); LN was sometimes replicated in dimension 2 ([VC-WM]: 52.8%) and dimension 1 ([VS-FR]: 36.4%). These three subtest scores were multidimensional, and hence had cross-loadings.

**Figure 4:**
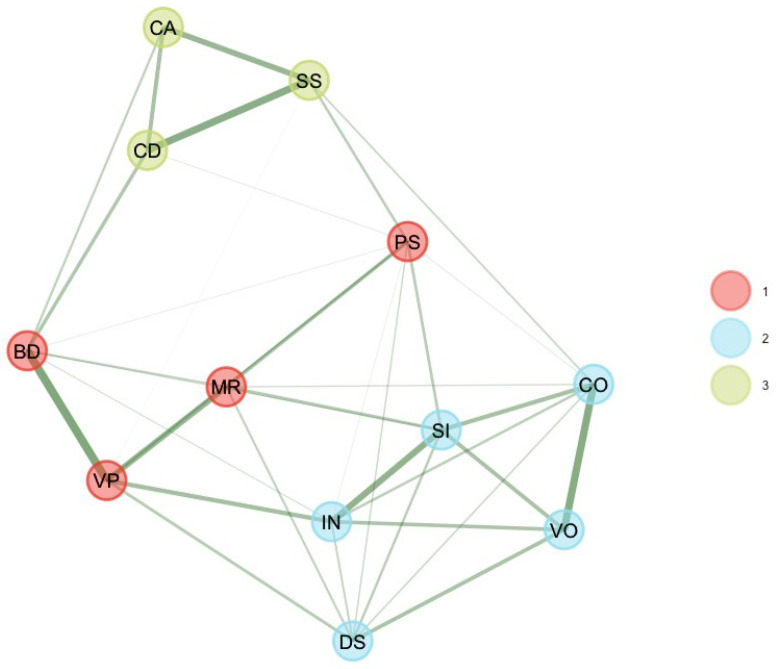
Final bootEGA network for 12–13 years.

Finally, the three unstable subtest scores were removed, and EGA and bootEGA were re-estimated. EGA and bootEGA showed three dimensions (see Table 1 and Figure 4). Dimension 1 represents a mixture of visual–spatial (BD, VP) with one fluid reasoning (MR) and one working memory subtest score (PS). The second dimension represents verbal comprehension (SI, VO, IN, CO) with Digit Span (DS). Finally, the third dimension is processing speed (CD, SS, CA). Frequencies indicate that three dimensions were found 89.6% of the time of bootstrap replicates while two dimensions were found 8% of the time of bootstrap replicates. Regarding the structural consistency, results indicate that dimension 3 [PS] and dimension 1 [VC + DS] were stable (respectively, 97.4% and 89%). Dimension 2 [VS-FR-WM] was still unstable (69.2%). However, item stability values across each dimension in the bootstrap samples indicate that all subtest scores values were higher than 0.70; hence, all subtest scores were stable.

### 3.5. Ages 14 to 16 Years Network Analyses

EGA with the GLASSO estimation and Walktrap community detection algorithm estimated three dimensions (Table 1). A first dimension represents a mixture of visual–spatial ([VS]; BD, VP), fluid reasoning ([FR]; MR, FW, AR), and working memory ([WM]; DS, PS, LN). The second dimension is consistent with verbal comprehension ([VC]; SI, VO, IN, CO), while the third dimension represents processing speed ([PS]; CD, SS, CA).

Parametric bootEGA with 500 iterations for the age group 14–16 estimated a typical structure (median structure) with four dimensions. A first dimension represents a mixture of visual–spatial (BD, VP), fluid reasoning (MR, FW, AR), and Picture Span (PS). The second dimension is consistent with working memory (DS, LN). The third dimension represents processing speed (CD, SS, CA), while the fourth dimension is consistent with verbal comprehension (SI, VO, IN, CO). Frequencies indicate that four dimensions were found 49.4% of the time of bootstrap replicates, and three dimensions were found 47.6% of the time. These results suggest that the three to four dimensions solutions were relatively unstable.

Then, structural consistency indicates that dimensions 2 [VC] and 3 [PS] were stable (respectively, 97% and 96.4%). Dimension 1 [VS-FR-WM] was unstable (31.4%). This finding is partially consistent with item stability analysis. Results indicate that three subtest scores values were below 0.70 and hence unstable (DS, LN, PS). Most of the unstable subtest scores belonged to dimension 1.

Regarding these unstable subtest scores, item stability values across each dimension in the bootstrap samples indicate that DS was replicated in dimension 1 ([VS-FR]: 65.8%) and dimension 4 ([WM]: 33.2%); LN was replicated in dimension 1 ([VS-FR]: 64.8%) and dimension 4 ([WM]: 33%); Picture Span (PS) was sometimes replicated in dimension 1 (57.8%), in dimension 3 ([PS]: 21.8%), and in dimension 4 (20%). These three subtest scores are multidimensional, and hence, have cross-loadings.

**Figure 5:**
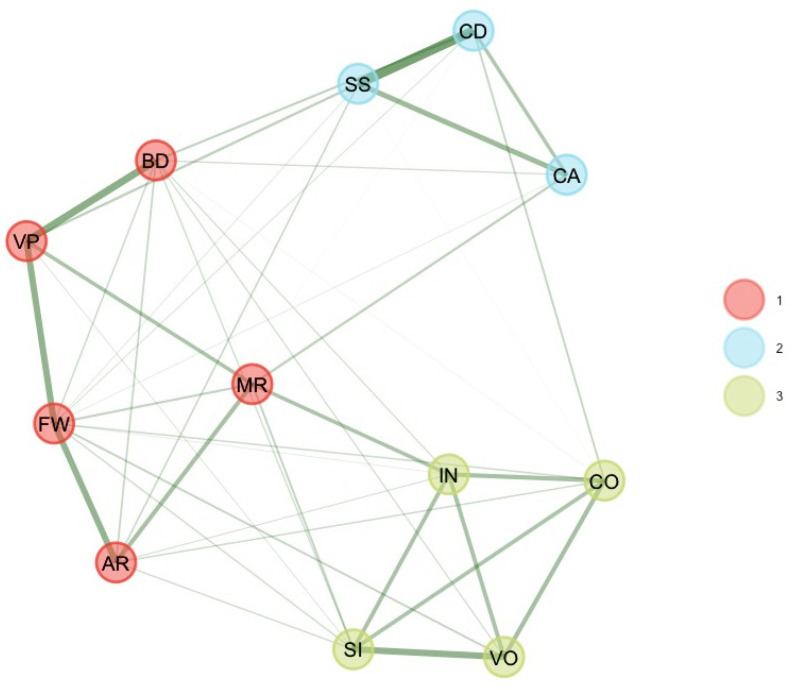
Final bootEGA network for 14–16 years.

Finally, these three unstable subtest scores were removed, and EGA and bootEGA were re-estimated. EGA and bootEGA suggested three dimensions (see Table 1 and Figure 5). Dimension 1 represents a mixture of visual–spatial (BD, VP), fluid reasoning (MR, FW, AR), while dimension 2 represents processing speed (CD, SS, CA). The third dimension is consistent with verbal comprehension (SI, VO, IN, CO). Frequencies indicate that three dimensions were found 91.2% of the time of bootstrap replicates and four dimensions were found 3.6% of the time. Concerning the structural consistency, results indicate that all dimensions were very stable (dimension 1 [VS-FR] = 85.8%; dimension 2 [VC] = 93.8%; and dimension 3 [PS] = 95.4%). This finding is consistent with item stability analysis. Item stability values across each dimension in the bootstrap samples indicate that all subtest scores values were higher than 0.70; hence, all subtest scores were stable. Removing these three subtest scores improved the stability of the dimensions.

### 3.6. All Age Groups Network Analysis

**Figure 6:**
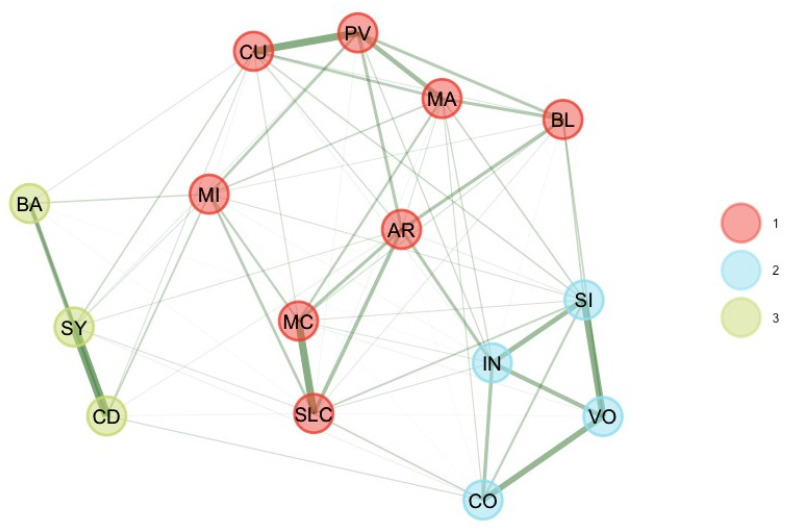
Final bootEGA network for the total sample.

Because previous results could be due to the small sample size of each age subgroups (Isvoranu and Epskamp 2021; Isvoranu et al. 2022), we run EGA and bootEGA for the total sample (N = 1049). bootEGA identified three clusters (Table 1 and Figure 6). A first cluster represents a mixture of visual–spatial (BD, VP), fluid reasoning (MR, FW, AR), and working memory (DS, PS, LN). The second dimension is consistent with verbal comprehension (SI, VO, IN, CO), while the third dimension represents processing speed (CD, SS, CA). Frequencies indicate that three dimensions were found 89.2% of the time of bootstrap replicates. Moreover, results indicate that all subtest scores values were higher than 0.70 and hence stable (FW, PS, AR, DS, LN, CA). Concerning the structural consistency, results indicate that all dimensions were very stable (dimension 1 [VS-FR] = 88.8%; dimension 2 [VC] = 100%, and dimension 3 [PS] = 100%).

## 4. Discussion

Within the NP approach, Golino and Epskamp (2017) proposed exploratory graph analysis (EGA) as a new procedure to determine the number of dimensions in psychological instruments. Thus, the first goal of the present paper was to determine the number of dimensions in the WISC–V^FR^ with five standardization sample age groups (6–7, 8–9, 10–11, 12–13, and 14–16 years).

For all age groups, EGA with the GLASSO network estimation method and the Walktrap community detection algorithm estimated three dimensions, except for 8–9 years for whom a four-dimension structure was identified (see Table 1). First, EGA provided evidence for the robustness of the processing speed dimension [PS–Gs] with strong association between Coding (CD), Symbol Search (SS), and Cancellation (CA). Second, strong relations between Similarities (SI), Vocabulary (VO), Information (IN), and Comprehension (CO) were observed for all age groups, supporting the verbal comprehension dimension [VC–Gc]. Third, the visual–spatial subtest scores (BD, VP) and the fluid reasoning subtest scores (MR, FW) were strongly associated in all age groups. This finding is not consistent with the distinction made by the publisher of the WISC–V^FR^ between the visual–spatial and fluid reasoning index scores. This dimension is more aligned with the “old” perceptual reasoning factor [PR]. The lack of distinction between visual–spatial and fluid reasoning is also consistent with EFA and CFA conducted on the WISC–V^FR^ by Lecerf and Canivez (2018, 2022), the findings from the US WISC–V (Canivez et al. 2018; Dombrowski et al. 2018, 2021), the UK WISC–V (Canivez et al. 2019), the German WISC–V (Pauls and Daseking 2021), and the Scandinavian WISC–V (Egeland et al. 2022). Fourth, a separate working memory cluster was only observed for ages 8–9 years, with strong associations between Digit Span (DS), Picture Span (PS), Letter-Number (LN), and Arithmetic (AR) subtest scores. For the other sample age groups, working memory subtest scores were associated with the perceptual reasoning dimension (6–7, 10–11, 12–13, and 14–16 years) and/or with the verbal comprehension dimension (6–7, 10–11, and 12–13 years). In sum, and most importantly, these findings are not consistent with the five-factor structure suggested by the publisher of the WISC–V^FR^.

However, because random sampling variability and/or parameter estimation variability influence the stability of the results, we examined the reproducibility of EGA results with bootstrap exploratory graph analysis (bootEGA) developed by Christensen and Golino (2021b). This method allows the investigation of the stability and robustness of the dimensions identified by EGA. bootEGA allows the estimation of the structural consistency and *item stability*. Indeed, the variability can conduct to a dimensional structure that does not generalize to other samples. Therefore, the number and the content of the dimensions can be inconsistent across replicate samples. Thus, the second goal of the present paper was to examine the stability and the robustness of the number of dimensions identified by EGA in the WISC–V^FR^ with five standardization sample age groups (6–7, 8–9, 10–11, 12–13, and 14–16 years) by using bootEGA. We applied bootEGA for 500 iterations.

Parametric bootEGA results indicate that the median structure had three dimensions for ages 6–7, 10–11, and 12–13 years, and four dimensions for ages 8–9 and 14–16 years. bootEGA results are consistent with EGA’s results for ages 6–7, 8–9, 10–11, and 12–13 years. For ages 14–16 years, bootEGA suggested four dimensions while EGA identified three dimensions only. However, the confidence interval was relatively large for the 14–16 age group (2.85–5.15). These typical three-dimension to four-dimension solutions are different from the five-factor structure based on CFA reported by the publisher of the WISC–V^FR^.

The next step concerned the frequency of each dimension solution across all bootstrap replicate samples, which shows important instability. These results suggest that the three-dimension solution might be stable only for the ages of 12–13 years (81.8%). In other words, three dimensions were found 81.8% of the time of 500 bootstrap replicates for this age sample. For ages 6–7 and 10–11 years, the three-dimension solution was more frequent but unstable (respectively, 55% and 62%). For ages 8–9 and 14–16 years, the four-dimension solution was more frequent but unstable (respectively, 54.2% and 49.4%). Overall, three-dimension to four-dimension solutions were not stable enough. Most importantly, the frequencies do not support the five-factor structure based on CFA proposed by the publisher of the WISC–V^FR^.

Therefore, we examined the structural consistency, which indicates how often the EGA dimension is exactly replicated across all bootstrap replicate samples. The structural consistency allows the determination of which dimensions are particularly unstable, in a multidimensional context. This indicator can be considered as an internal consistency measure and represents the proportion of time each dimension is exactly found. The structural consistency is an indicator of the construct validity. If we consider that acceptable structural consistency is ≥0.75 (75%), then results indicate that the processing speed [PS] dimension was stable for all age groups, except for 6–7 years. In addition, results indicate that the verbal comprehension [VC] dimension was stable for ages 8–9 and 14–16 years. It means that all other dimensions presented low structural consistency, and hence, are not reflecting homogenous dimensions.

Because most of the dimensions were not stable enough, we examined item stability. This statistic indicates how often a subtest replicates in the dimension identified by EGA, but also in the other dimensions in the replicate networks. This statistic is useful to determine which subtest score causes the instability of the dimension and lowers the structural consistency. We consider that subtest stability is appropriate if the subtest replicates at least 70% of the time in its EGA dimension (≥0.70). The subtest stability analysis allows the determination of which subtests to retain to increase the dimension’s structural consistency. Results indicate that most subtest scores replicated adequately in the dimensions proposed by EGA. However, six subtest scores presented low *item stability* for some age groups. These subtest scores are Picture Span (PS: 6–7, 8–9, 10–11, and 14–16 years), Digit Span (DS: 6–7, 8–9, and 14–16 years), Arithmetic (AR: 6–7, 8–9, and 12–13 years), Letter-Number (LN: 6–7, 8–9, 12–13, and 14–16 years), Figure Weights (FW: 6–7 and 12–13 years), and Cancellation (CA: 6–7 years). These subtest scores are multidimensional. Regarding Arithmetic, results support the hypothesis that Arithmetic is a mixture of perceptual reasoning (6–7, 8–9, and 12–13 years), verbal comprehension (6–7 and 12–13 years) and working memory (6–7 and 8–9 years).

Because the main purpose when administering the WISC–V^FR^ is to interpret homogenous scores, EGA and bootEGA were re-estimated after removing the subtests with the lowest item stability within each age group. For all age groups, the final EGA and bootEGA dimensionality structures had three dimensions only (see Table 1). Removing the subtest scores with low *item stability* improved the dimensions’ structural consistency. The three-dimension solution was much more stable (≥0.75) for all age groups, except for 10–11 years (68.6%). All subtest scores had item stability >0.70, indicating that all subtest scores were replicating in their respective EGA dimension. A structure with four dimensions occurred from 0.2% (6–7) to 25.4% (10–11 years). A five-dimension solution was observed 2.4% in ages 10–11 years. The first dimension includes Coding, Symbol Search, and Cancellation in all age groups, except in ages 6–7 for whom Cancellation was removed. This first dimension represents processing speed [PS]. The second dimension corresponds to verbal comprehension and includes Similarities (SI), Vocabulary (VO), Information (IN), and Comprehension (CO). It should be noted that for ages 12–13, Digit Span (DS) was associated with the four verbal tasks. The third dimension represents the “old” perceptual reasoning factor [PR] and shows strong association between Block Design (BD), Visual Puzzles (VP), and Matrix Reasoning (MR) for all age groups. This dimension was associated with Figure Weights (FW) for ages 8–9, 10–11, and 14–16 years. Arithmetic (AR) was also associated with the PR dimension for ages 10–11 and 14–16 years. Finally, Digit Span (DS) and Letter-Number (LN) were associated with PR for ages 10–11, while Picture Span (PS) was associated with the PR dimensions for ages 12–13.

EGA and bootEGA results are not completely consistent with those obtained with exploratory factor analyses (EFA) conducted on the WISC–V^FR^ with the total sample (Lecerf and Canivez 2018) and the five standardization sample age groups (Lecerf and Canivez 2022). In both EFA studies, results indicated that the general factor of intelligence accounted for the most important part of the common variance and that the contributions of the factors (i.e., the broad abilities: VCI, VSI, etc.) were weak. Although EGA and EFA pursue the same goal, estimating the number of dimensions, they rely on different statistical procedures. With EGA and bootEGA, the edges (the links) in the network are partial correlation coefficients. Therefore, edges quantify the relation between two subtest scores after controlling for all other subtest scores in the model. In consequence, the psychometric general variance associated with all subtest scores is removed in the network (McGrew 2023). Thus, network analyses look like bifactor models. In contrast, EFA uses zero-order correlations instead of partial correlations. Therefore, EFA analyses the total common variance between all variables. Taken together, EGA and EFA are complementary and suggest that the interpretation of the WISC–V^FR^ should be based on the FSIQ (as a proxy of the *g*-factor) and only three dimensions: verbal comprehension (SI-VO-IN-CO), processing speed (CD, SS, CA) and a mixture of visuospatial (BD, VP), reasoning (MR, FW, AR), and working memory (DS, PS, LN). Only two broad CHC scores are supported when the large psychometric *g* variance is removed: VCI and PSI. bootEGA did not support the interpretation of the distinct broad abilities Gf (fluid reasoning), Gv (visual–spatial) and Gwm (working memory). The mixture Gf–Gv–Gwm is consistent with many studies that have shown the overlap between Gf and working memory (Ghisletta and Lecerf 2023). This mixture might represent attention control with maintenance and disengagement.

Clinical utility is aimed at assessing whether the test’s scores improve the clinical decision and is related to construct validity (i.e., diagnoses, interventions, etc.) (Canivez et al. 2019; Hunsley and Mash 2007). Like the four other sources of evidence, internal validity provides information regarding construct validity, and hence, regarding test use and interpretation (AERA 2014; Hughes 2018; Kane 2013). Assessment of test score validity might be done by exploratory and/or confirmatory analyses. The publisher of the WISC–V^FR^ examined the factorial structure through confirmatory factor analysis only. However, we agree with Beaujean (2015) that when a test is revised so that content, scores and/or theory have changed, exploratory analyses are more appropriate than confirmatory ones. Substantial modifications were introduced between the WISC–IV^FR^ and the WISC–V^FR^. Therefore, it seems necessary to examine the structural validity of the WISC–V^FR^ using exploratory analyses instead of confirmatory ones (Lecerf and Canivez 2018, 2022). That is the reason why we considered that EGA and bootEGA were useful to assess the construct validity of the WISC–V^FR^ subtest scores to support the interpretation and the use of these subtest scores. Finally, it should be noted that from our point of view, exploratory analyses can be conducted from a confirmatory perspective. Based on the factorial structure proposed by the publisher of the WISC–V^FR^, five clusters were expected. However, after removing the large *g* variance, the presence of only two–three broad CHC abilities is validated, because we consider the statistical model as a theoretical model.

## 5. Limitations of the Present Study

This study contains several limitations. First, the data are the correlation matrices reported in the French interpretive manual for the five age groups (Wechsler 2016). Second, these correlations have only two decimals. However, Lecerf and Canivez (2018, 2022) reported EFA and CFA results, such as those reported in the WISC–V^FR^ *interpretive manual*. Third, these correlation matrices are cross-sectional rather than longitudinal, which does not allow for really testing the mutualism model proposed by van der Maas et al. (2006).

## 6. Conclusions

EGA and bootEGA represent new tools to assess the construct validity of psychological instruments, such as the WISC–V^FR^. EGA and bootEGA supported a three-dimension solution with any of the five WISC–V^FR^ age groups. EGA and bootEGA results suggested that the five-factor higher-order model preferred by the WISC–V^FR^ publisher might be overfactored. From a practical point of view, the present results have several very important implications for the use and the interpretation of the WISC–V^FR^ subtest scores. The five-factor higher-order model preferred by the publisher is not adequate across the five age groups. After removing unstable subtest scores, the final dimensionality structure has three dimensions only: verbal comprehension [VC], processing speed [PS], and perceptual reasoning [PR]. EGA and bootEGA do not support the distinction between [VS] and [FR] in any of the five age groups. Furthermore, the present finding suggests that the WM index is not appropriate because DS, PS, and LN are mostly unstable, and/or associated with other dimensions. The present results suggest that interpretation of the WISC–V^FR^ scores should focus on VC (SI, VO, IN, CO), PS (CD, SS, CA), and PR (BD, VP, MR), which are stable dimensions. To interpret these three broad abilities, practitioners must create three new extended composite scores. However, these three broad abilities might not be latent attributes, but emerge from the interactions between cognitive processes. The new composite scores will not estimate a single attribute but represent summary statistics.

## Figures and Tables

**Table 1:** Main EGA and bootEGA results for the five age groups and the total sample.

	EGA	bootEGA	Final bootEGA (Unstable Subtests Removed)
6–7 years	Three clusters: - VS (BD, VP) + FR (MR, FW, AR) + WM (DS, PS) - VC (SI, VO, IN, CO) + LN - PS (CD, SS, CA)	Three clusters: - VS (BD, VP) + FR (MR, FW, AR) + WM (DS, PS) - VC (SI, VO, IN, CO) + LN - PS (CD, SS, CA)	Three clusters: - VC (SI, VO, IN, CO) - PR (BD, VP, MR) - PS (CD, SS)
8–9 years	Four clusters: - VS (BD, VP) + FR (MR, FW) - WM (DS, PS, LN) + AR - VC (SI, VO, IN, CO) - PS (CD, SS, CA)	Four clusters: - VS (BD, VP) + FR (MR, FW) - WM (DS, PS, LN) + AR - VC (SI, VO, IN, CO) - PS (CD, SS, CA)	Three clusters: - PR (BD, VP, MR, FW) - VC (SI, VO, IN, CO) - PS (CD, SS, CA)
10–11 years	Three clusters: - VS (BD, VP) + FR (MR, FW, AR) + WM (DS, LN) - VC (SI, VO, IN, CO) + PS - PS (CD, SS, CA)	Three clusters: - VC (SI, VO, IN, CO) + PS - PS (CD, SS, CA) - VS (BD, VP) + FR (MR, FW, AR) + WM (DS, LN)	Three clusters: - VC (SI, VO, IN, CO) - PS (CD, SS, CA) - PR + WM (BD, VP, MR, FW, DS, LN, AR)
12–13 years	Three clusters: - VS (BD, VP) + FR (MR, FW, AR) + PS - VC (SI, VO, IN, CO) + WM (DS, LN) - PS (CD, SS, CA)	Three clusters: - VC (SI, VO, IN, CO) + WM (DS, LN) - VS (BD, VP) + FR (MR, FW, AR) + PS - PS (CD, SS, CA)	Three clusters: - PR + FR + WM (BD, VP, MR, PS) - VC (SI, VO, IN, CO) + DS - PS (CD, SS, CA)
14–16 years	Three clusters: - VS (BD, VP) + FR (MR, FW, AR) + WM (DS, PS, LN) - VC (SI, VO, IN, CO) - PS (CD, SS, CA)	Four clusters: - VS (BD, VP) + FR (MR, FW, AR) + PS - WM (DS, LN) + AR - PS (CD, SS, CA) - VC (SI, VO, IN, CO)	Three clusters: - VS (BD, VP) + FR (MR, FW, AR) - PS (CD, SS, CA) - VC (SI, VO, IN, CO)
All age groups	Three clusters: - VS (BD, VP) + FR (MR, FW, AR) + WM (DS, PS, LN) - VC (SI, VO, IN, CO) - PS (CD, SS, CA)	Three clusters: - VS (BD, VP) + FR (MR, FW, AR) + WM (DS, PS, LN) - VC (SI, VO, IN, CO) - PS (CD, SS, CA)	Three clusters: - VC (SI, VO, IN, CO) - PS (CD, SS, CA) - VS (BD, VP) + FR (MR, FW, AR) + WM, (DS, PS, LN)

Note: PR (perceptual reasoning) = VS + FR, the “old” perceptual reasoning factor of the WISC-IV which represents the visual-spatial and fluid reasoning index scores in the WISC-V.

## Data Availability

Data (correlation matrices) is available in the WISC-V^FR^ interpretive manual and is copyrighted. No new data were created or analyzed in this study. Data sharing is not applicable to this article.

## Notes

1. Factor can be defined as an unobservable variable that influences one or more observed variables. Factor accounts for the correlations between observed variables.

2. A “bond” corresponds to a “unit”, an “element”.

3. We can consider that the process overlap theory (POT) proposed by Kovacs and Conway (2016) is compatible with a NP approach.

4. There are two ways to interpret the relation between latent and manifest variables: a reflective (causal) one and a formative one.

5. It should be noted that network models and latent variable models can be combined (Epskamp et al. 2017).

6. If λ = 0, there is no penalization.

7. Non-parametric bootstrap algorithm is not available with correlation matrix.

8. Only values ≥0.15 were reported.
